# Pien Tze Huang Overcomes Multidrug Resistance and Epithelial-Mesenchymal Transition in Human Colorectal Carcinoma Cells via Suppression of TGF-*β* Pathway

**DOI:** 10.1155/2014/679436

**Published:** 2014-11-19

**Authors:** Aling Shen, Hongwei Chen, Youqin Chen, Jiumao Lin, Wei Lin, Liya Liu, Thomas J. Sferra, Jun Peng

**Affiliations:** ^1^Academy of Integrative Medicine, Fujian University of Traditional Chinese Medicine, 1 Huatuo Road, Minhou Shangjie, Fuzhou, Fujian 350122, China; ^2^Fujian Key Laboratory of Integrative Medicine on Geriatrics, Fujian University of Traditional Chinese Medicine, 1 Huatuo Road, Minhou Shangjie, Fuzhou, Fujian 350122, China; ^3^Rainbow Babies & Children's Hospital, Case Western Reserve University School of Medicine, 11100 Euclid Avenue, Cleveland, OH 44106, USA; ^4^Postdoctoral Workstation, Zhangzhou Pien Tze Huang Pharmaceutical Co., Ltd., Shangjie, Zhangzhou, Fujian 363000, China

## Abstract

The traditional Chinese medicine formula Pien Tze Huang (PZH) has long been used as a folk remedy for cancer. To elucidate the mode of action of PZH against cancer, in the present study we used a 5-FU resistant human colorectal carcinoma cell line (HCT-8/5-FU) to evaluate the effects of PZH on multidrug resistance (MDR) and epithelial-mesenchymal transition (EMT) as well as the activation of TGF-*β* pathway. We found that PZH dose-dependently inhibited the viability of HCT-8/5-FU cells which were insensitive to treatment of 5-FU and ADM, demonstrating the ability of PZH to overcome chemoresistance. Furthermore, PZH increased the intercellular accumulation of Rhodamine-123 and downregulated the expression of ABCG2 in HCT-8/5-FU cells. In addition, drug resistance induced the process of EMT in HCT-8 cells as evidenced by EMT-related morphological changes and alteration in the expression of EMT-regulatory factors, which however was neutralized by PZH treatment. Moreover, PZH inhibited MDR/EMT-enhanced migration and invasion capabilities of HCT-8 cells in a dose-dependent manner and suppressed MDR-induced activation of TGF-*β* signaling in HCT-8/5-FU cells. Taken together, our study suggests that PZH can effectively overcome MDR and inhibit EMT in human colorectal carcinoma cells via suppression of the TGF-*β* pathway.

## 1. Introduction

Colorectal cancer (CRC) is a serious public health problem, with more than one million new cases and over a half million deaths worldwide each year [1, 2]. Although surgical resection to completely remove the cancer offers the best prognosis for long-term survival, a substantial portion of CRC patients is not suitable for surgery because of the presenting of metastasis at the time of diagnosis; and surgery cannot always extirpate cancer recurrence [3, 4]. Therefore, chemotherapy, especially 5-fluorouracil- (5-FU-) based regimens, remains an important therapeutic option for advanced CRC. However, due to multidrug resistance and an unacceptable level of toxicity against normal cells, systemic chemotherapy using 5-FU-based regimens produces objective response rates of only 10–20% [5–8]. These problems highlight the urgent need for the development of novel therapeutic strategies and agents.

Multidrug resistance (MDR), the cellular resistance to numerous drugs differing in mechanisms of action and/or chemical structures, is a major cause of failure of cancer chemotherapy. MDR is mediated by multiple mechanisms, including overexpression of energy-dependent transporters that eject anticancer drugs from cells and acquisition of epithelial-mesenchymal transition (EMT) [9–12]. ATP-binding cassette (ABC) family of transporter proteins can pump various xenobiotics out of the cell, reducing the intracellular accumulation of chemotherapeutic drugs [13, 14]. As a half-transporter of the G subfamily of ABC transporter, breast cancer resistance protein (BCRP/ABCG2) is known to play a crucial role in multidrug resistance. The overexpression of ABCG2 protects cells from xenobiotic- and toxin-induced damages by increasing efflux of these compounds [15]. Thus, inhibition of ABC transporter activity is a potential approach to overcome the chemoresistance [16].

EMT is a biological process in which epithelial cells lose their polarity and cell-cell adhesion, and acquire migratory and invasive properties of mesenchymal cells. The process of EMT is observed during embryonic development, wound healing, organ fibrosis, and cancer progression and metastasis [17–21]. Epithelial and mesenchymal cells are different in both phenotype and function. Epithelial cells have an apical-basal polarity, express high levels of epithelial markers such as E-cadherin, and are closely connected to each other forming epithelial adherent junctions. In contrast, mesenchymal cells lack the cell polarity, highly express mesenchymal markers such as N-cadherin and vimentin, display a spindle-shaped morphology, and interact with each other through focal points [17]. After acquiring a mesenchymal phenotype through the process of EMT, carcinoma cells obtain the capacities to invade adjacent tissues, break through the basement membrane, and eventually enter the bloodstream [22–24]. Furthermore, accumulating evidence has shown that the process of EMT is also strongly associated with MDR in various types of human malignancies including CRC [25–34]. Thus, EMT not only confers cancer cells, the unique advantage of migration and invasion, leading to cancer progression and metastasis, but also plays an important role in drug resistance, resulting in the failure of clinical chemotherapies.

EMT in cancer progression and metastasis is highly regulated by a diverse array of cytokines and growth factors. Prominent among these regulatory factors is the transforming growth factor *β* (TGF-*β*) superfamily [12, 34, 35] that consists of TGF-*β* proteins, bone morphogenetic proteins (BMPs), growth differentiation factors (GDFs), and various other polypeptide morphogens [36]. The prototypic member of this superfamily is TGF-*β*1. The activation of TGF-*β* signaling pathway is initiated by the binding of ligands to a type II receptor, which recruits, phosphorylates, and activates a type I receptor. The activated type I receptor then phosphorylates receptor-regulated SMADs (R-SMADs, e.g., SMAD2/3) that in turn bind the coSMAD (e.g., SMAD4). The R-SMAD/coSMAD complex translocates to the nucleus to regulate the expression of target genes, including three families of transcription factors, Snail, ZEB, and bHLH families [37, 38]. Upon activation, these transcription factors suppressed epithelial marker gene expression and upregulated mesenchymal gene expression leading to EMT [35]. Thus, inhibiting EMT via suppression of TGF-*β* may represent a novel therapeutic strategy for reversing MDR.

Natural products, including traditional Chinese medicines (TCM), have recently received great interest since they have relatively few side-effects as compared to modern chemotherapeutics and have long been used to treat various diseases including cancer [39, 40]. TCM formula is a complex combination of many natural products, each of which contains numerous chemical compounds. TCM formulas therefore are considered to be multicomponent and multitarget agents that may exert their therapeutic activities in a more holistic way. Pien Tze Huang (PZH) is a well-known TCM formula prescribed by a royal physician more than 450 years ago during the Chinese Ming Dynasty [41]. PZH has also been used in China and Southeast Asia for centuries as a folk remedy for various cancers. Previously, we reported that PZH inhibits colorectal cancer growth both* in vivo* and* in vitro* via promotion of cancer cell apoptosis, inhibition of cell proliferation, and tumor angiogenesis [42–47]. To further elucidate the mechanism of its antitumor activities, in the present study we evaluated the effects of PZH on MDR and EMT in a 5-FU resistant human colorectal carcinoma cell line and investigated the underlying mechanisms of its action.

## 2. Methods

### 2.1. Materials and Reagents

Roswell Park Memorial Institute (RPMI) 1640 medium, fetal bovine serum (FBS), penicillin-streptomycin, TRIzol reagent, and Rhodamine-123 were obtained from Life Technologies Corporation (Grand Island, NY, USA). N-cadherin, E-cadherin antibodies were purchased from Abcam (HK) Ltd. (Hongkong, China). TGF-*β*, SMAD4, ZEB1, ZEB2, and *β*-actin antibodies and horseradish peroxidase- (HRP-) conjugated secondary antibodies were provided by Cell Signaling Technology (Beverly, MA, USA). ABCG2 antibody was purchased from Sangon Biotech (Shanghai, China). Transwell chambers were obtained from Corning Life Sciences (Corning, NY, USA). BD BioCoat Matrigel Invasion Chamber was purchased from BD Bioscience (San Jose, CA, USA). PrimeScript RT reagent Kit was provided by Takara Biotechnology (Dalian) Co., Ltd. (Dalian, Liaoning, China). All the other chemicals, unless otherwise stated, were obtained from Sigma Chemicals (St. Louis, MO, USA).

### 2.2. Preparations of PZH

PZH was obtained from and authenticated by the sole manufacturer Zhangzhou Pien Tze Huang Pharmaceutical Company Limited, China (Chinese FDA approval number: Z35020242). Stock solutions of PZH were prepared just before use by dissolving the PZH powder in PBS (phosphate buffered saline) to a concentration of 20 mg/mL. The working concentrations of PZH were made by diluting the stock solution in the culture medium.

### 2.3. Cell Culture

Human colorectal carcinoma HCT-8 cell and 5-FU resistant HCT-8/5-FU cells were obtained from Nanjing KeyGen Biotech. Co. Ltd. (Nanjing, Jiangsu, China). Cells were grown in RPMI 1640 medium containing 10% (v/v) FBS, 100 units/mL penicillin, and 100 *μ*g/mL streptomycin in a 37°C humidified incubator with 5% CO_2_. The cells were subcultured at 80–90% confluency. HCT-8/5-FU cells were cultured in RPMI-1640 containing 15 *μ*g/mL of 5-FU.

### 2.4. Evaluation of Cell Viability by MTT Assay

Viability of HCT-8 cells and HCT-8/5-FU cells was examined by the 3-(4, 5-dimethylthiazol-2-yl)-2, 5-diphenyltetrazolium bromide (MTT) colorimetric assay. Cells were seeded into 96-well plates at a density of 6 × 10^3^ cells/well in 0.1 mL medium and then treated with various concentrations of 5-FU, Adriamycin (ADM), or PZH for indicated periods of time. 100 *μ*L MTT (0.5 mg/mL in PBS) was added to each well, and the samples were incubated for an additional 4 h at 37°C. The purple-blue MTT formazan precipitate was dissolved in 100 *μ*L DMSO. The absorbance was measured at 570 nm using an ELISA reader (BioTek, Model ELX800, USA).

### 2.5. Observation of Morphological Changes

HCT-8 cells and HCT-8/5-FU cells were seeded into 6-well plates at a density of 5 × 10^5^ cells/well in 2 mL medium. The cell morphology was observed by using a phase-contrast microscope (Leica, German). The photographs were taken at a magnification of ×400.

### 2.6. Measurement of Rhodamine-123 Accumulation

HCT-8/5-FU cells were seeded in 6-well plates at a density of 2.5 × 10^5^ cells/mL in 2 mL medium, and then the cells were treated with indicated concentration of PZH (0–0.75 mg/mL) for 24 h. 1 × 10^6^ cells were resuspended in 1 mL medium with 5 *μ*g/mL Rhodamine-123 and incubated for an additional 10 min at 37°C with 5% CO_2_. The accumulation of Rhodamine-123 was stopped by cooling on ice and cells were washed in ice-cold PBS before fluorescence-activated cell sorting (FACS) analysis. The accumulation of Rhodamine-123 was present as relative values.

### 2.7. Measurement of Cell Migration and Invasion by Transwell Assay

The migration assay and invasion assay were performed using transwell cell culture chambers, coated without (Corning Life Sciences) or with Matrigel Matrix (BD Biosciences). The inserts were placed within a 24-well chamber containing 0.7 mL RPMI-1640 with 10% fetal bovine serum as a chemoattractant. As before, HCT-8 and HCT-8/5-FU cells were seeded into 6-well plates and HCT-8/5-FU cells were treated with different concentrations of PZH for 24 h. Cells (5 × 10^4^ cells) were seeded into the inserts suspended in 0.2 mL of serum-free RPMI 1640. The cells were incubated at 37°C with 5% CO_2_ for 12 h or 24 h for migration and invasion assay. The upper surface of the filter was scraped to remove nonmigratory cells. Migrated and invaded cells were fixed and stained with crystal violet. For quantification, the average number of migrating cells per field was assessed by counting 3 random fields under a phase-contrast microscope (Leica, German) at a magnification of 200x.

### 2.8. RNA Extraction and RT-PCR Analysis

HCT-8 and HCT-8/5-FU cells (5 × 10^5^ of each) were seeded into 6-well plates in 2 mL medium and HCT-8/5-FU cells were treated with indicated concentrations of PZH for 24 h. Total RNA was isolated with TriZol Reagent. Oligo(dT)-primed RNA (1 *μ*g) was reverse-transcribed with PrimeScript RT reagent Kit according to the manufacturer's instructions. The obtained cDNA was used to determine the mRNA levels of ABCG2, N-cadherin, E-cadherin, TGF-*β*, SMAD4, ZEB1, and ZEB2 by PCR. GAPDH was used as an internal control. The primer sequences for all these genes were listed in Table 1.

### 2.9. Western Blot Analysis

HCT-8 cells and HCT-8/5-FU cells were seeded into 25 cm^2^ flasks at a density of 1.5 × 10^6^ cells/flask in 5 mL medium. HCT-8/5-FU cells were treated with the indicated concentrations of PZH for 24 h. The treated cells were lysed with mammalian cell lysis buffer containing protease and phosphatase inhibitor cocktails. Total protein concentrations were determined by BCA assay. Equal amounts of total proteins were resolved in 12% SDS-PAGE gels and electroblotted. The PVDF membranes were blocked with 5% skimmed milk and probed with primary antibodies ABCG2, N-cadherin, E-cadherin, TGF-*β*, SMAD4, ZEB1, ZEB2, and *β*-actin (1 : 1,000) overnight at 4°C and subsequently with the appropriate HRP-conjugated secondary antibody followed by enhanced chemiluminescence detection.

### 2.10. Statistical Analysis

All data are presented as the means of three determinations and were analyzed using the SPSS package for Windows (Version 18.0). Statistical analysis of the data was performed with Student's *t*-test and ANOVA. Differences with *P* < 0.05 were considered statistically significant.

## 3. Results

### 3.1. PZH Inhibits the Proliferation of 5-FU Resistant Human Colorectal Carcinoma Cell Line HCT-8/5-FU

**Figure 1:**
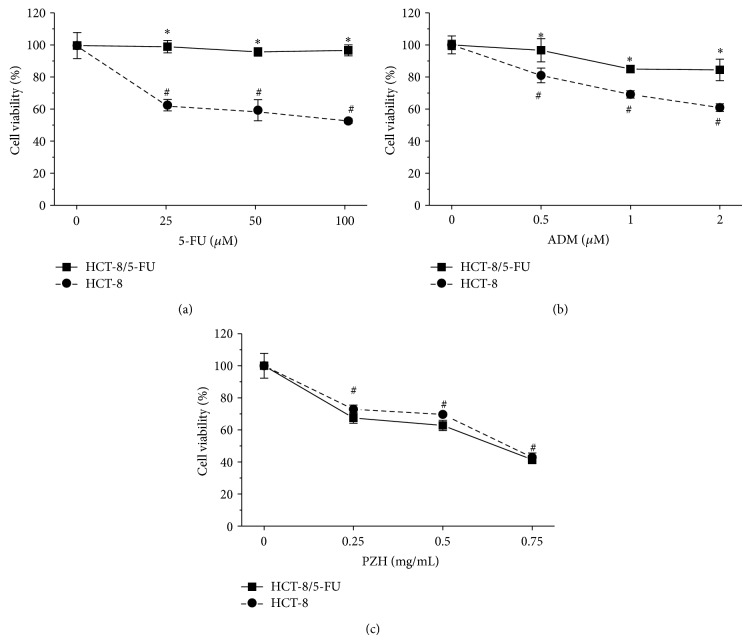
Effect of PZH on the viability of 5-FU resistant colorectal cancer cells. Cell viability was determined by MTT assay after HCT-8/5-FU and parental HCT-8 cells were treated with the indicated concentrations of 5-FU (a), ADM (b), and PZH (c) for 48 h. The data were normalized to the viability of control cells. Data are averages with S.D. (error bars) from at least three independent experiments. ^#^ *P* < 0.05, versus controls; ^*^ *P* < 0.05, versus HCT-8/5-FU cells.

To evaluate MDR activity in cancer cells and therapeutic effects of PZH, HCT-8/5-FU and parental cells were exposed to different concentrations of 5-FU, ADM, or PZH for 48 h and the MTT assay was performed to determine the cell viability. As shown in Figures 1(a) and 1(b), the viability of parental HCT-8 cells was significantly decreased by treatment with 5-FU or ADM, whereas HCT-8/5-FU cell viability did not remarkably change after 5-FU or ADM treatment, thus demonstrating the MDR properties of HCT-8/5-FU cells. However, administration of PZH significantly and dose-dependently reduced the viability of both parental and drug-resistant HCT-8 cells (Figure 1(c)), suggesting that PZH may possess the ability to overcome colorectal cancer cell chemoresistance.

### 3.2. PZH Inhibits Drug Efflux and ABCG2 Expression in HCT-8/5-FU Cells

**Figure 2:**
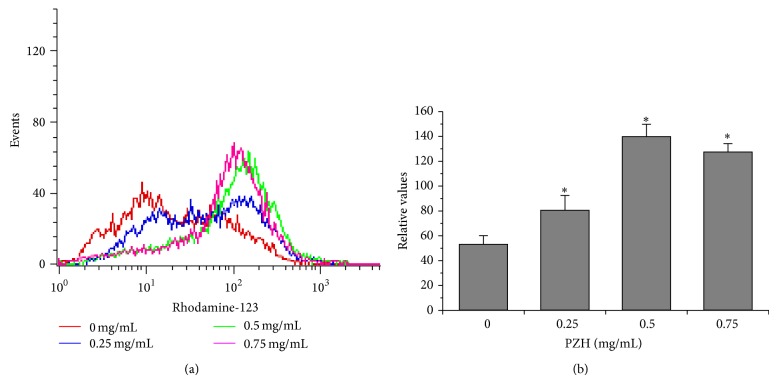
Effect of PZH on accumulation of Rhodamine-123 in HCT-8/5-FU cells. Intercellular accumulation of Rhodamine-123 was determined by FACS after treated with the indicated concentrations of PZH for 24 h (a). The accumulation of Rhodamine-123 present by relative values was averaged with S.D. (error bars) from at least three independent experiments and is summarized as histograms in (b). ^*^ *P* < 0.05, versus untreated HCT-8/5-FU cells.

**Figure 3:**
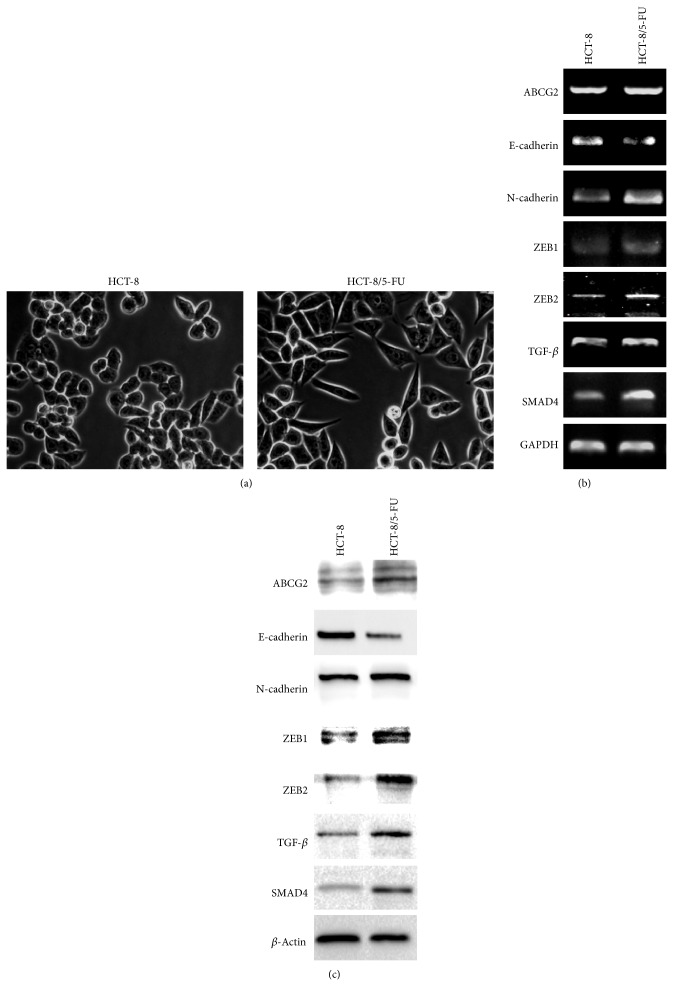
Effect of MDR on the morphological changes, the expression of ABCG2, EMT-related factors, and the activation of TGF-*β* pathway. (a) Morphology of HCT-8/5-FU and parental HCT-8 cells was observed using phase-contrast microscopy. The photographs were taken at a magnification of 400x. Images are representative of three independent experiments. (b-c) The mRNA and protein expression levels of ABCG2, E-cadherin, N-cadherin, ZEB1, ZEB2, TGF-*β*, and SMAD4 in parental HCT-8 and HCT-8/5-FU cells were determined by RT-PCR and Western Blot analyses. GAPDH or *β*-actin was used as the internal control for RT-PCR or Western Blot, respectively. Images are representatives of three independent experiments.

To evaluate the effect of PZH on drug efflux, Rhodamine-123 accumulation was determined as a measure of intracellular drug accumulation by FACS analysis. As shown in Figures 2(a) and 2(b), comparing with untreated control, PZH treatment increased the fluorescence intensity of Rhodamine-123, indicating that PZH is able to enhance the accumulation of Rhodamine-123 and suggesting that inhibition of drug efflux to increase the intercellular accumulation of chemotherapeutics might be one of the mechanisms for PZH to overcome MDR. To explore the underlying mechanism, we determined the expression of ABCG2 in HCT-8 cells and HCT-8/5-FU cells treated with or without PZH using RT-PCR and Western Blot analyses. As shown in Figures 3 and 6, drug resistance induced expression of ABCG2 was remarkably inhibited by PZH treatment.

### 3.3. PZH Inhibits MDR-Induced EMT in HCT-8/5-FU Cells

MDR-induced EMT was evaluated by observing the morphological changes in HCT-8/5-FU cells. As shown in Figure 3(a), compared with parental cells, 5-FU resistant HCT-8 cells (HCT-8/5-FU) displayed typical EMT morphological characteristics, including loss of cell polarity, spindle-shaped fibroblastoid-like morphology, and formation of pseudopodia. To further verify these results, we determined the expression of EMT-related factors in HCT-8/5-FU and parental cells using RT-PCR and Western Blot analyses. As shown in Figure 3, acquisition of drug resistance significantly decreased the mRNA and protein expression levels of epithelial marker E-cadherin, whereas those of mesenchymal markers such as N-cadherin, ZEB1, and ZEB2 were remarkably increased. However, PZH treatment profoundly neutralized the MDR-induced alteration in the expression of EMT-regulatory factors in both transcriptional and translational levels (Figure 6).

### 3.4. PZH Inhibits MDR-Enhanced Migration and Invasion of HCT-8/5-FU Cells

**Figure 4:**
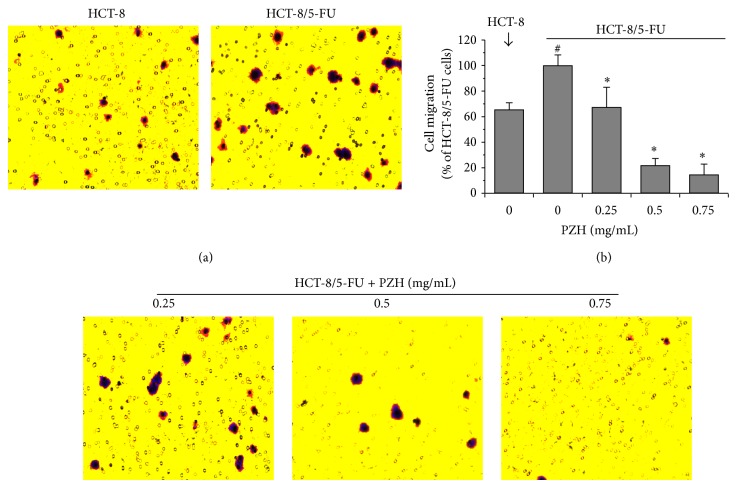
Effect of PZH on the migration of HCT-8/5-FU cells. HCT-8/5-FU cells were treated with indicated concentrations of PZH for 24 h. (a) The migration of HCT-8/5-FU or parental HCT-8 cells was determined using transwell cell culture chambers. Cells were stained with crystal violet; the photographs were taken at a magnification of 200x. (b) The average number of migrated cells was counted in 3 randomly selective fields. The data were normalized to the migration of HCT-8/5-FU cells (100%). Data are averages with S.D. (error bars) from three independent experiments. ^#^ *P* < 0.05, versus parental HCT-8 cells; ^*^ *P* < 0.05, versus HCT-8/5-FU cells without PZH treatment.

**Figure 5:**
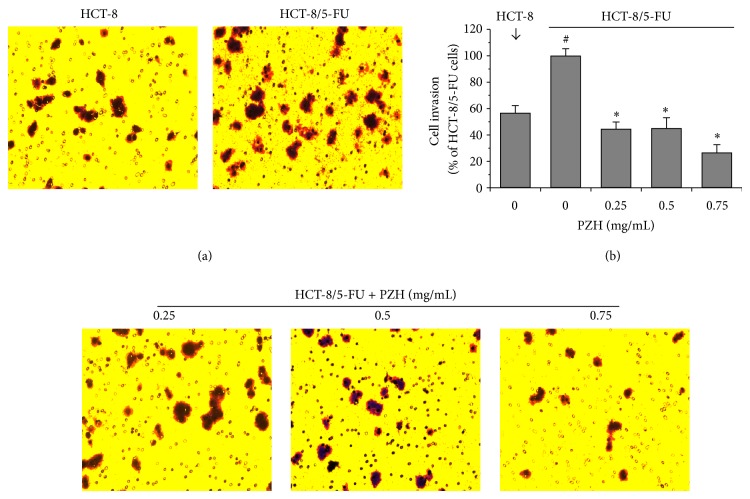
Effect of PZH on the invasion of HCT-8/5-FU cells. HCT-8/5-FU cells were treated with indicated concentrations of PZH for 24 h. (a) The invasion of HCT-8/5-FU or parental HCT-8 cells was determined using transwell cell culture chambers with membranes (8 *μ*M) coated with Matrigel matrix. Cells were stained with crystal violet; the photographs were taken at a magnification of 200x. (b) The average number of invaded cells was counted in 3 randomly selective fields. The data were normalized to the invasion of HCT-8/5-FU cells (100%). Data are averages with S.D. (error bars) from three independent experiments. ^#^ *P* < 0.05, versus parental HCT-8 cells; ^*^ *P* < 0.05, versus HCT-8/5-FU cells without PZH treatment.

Since MDR and/or EMT promote the metastasis of cancer cells, we next performed transwell assays to determine the effects of PZH on the migration and invasion of HCT-8/5-FU cells. As shown in Figure 4, acquisition of drug resistance significantly enhanced the migratory capacity of HCT-8 cells by approximately 52.8% (*P* < 0.05). However, treatment with 0.25–0.75 mg/mL of PZH dose-dependently reduced cell migratory of HCT-8/5-FU cells ability by 32.7%–85.5% (*P* < 0.05). In a consistent manner, PZH treatment inhibited MDR-enhanced invasion of colorectal cancer cells in a dose-dependent manner. As compared with untreated HCT-8/5-FU cells (100%), the invasive capacity of parental HCT-8 cells or HCT-8/5-FU cells treated with 0.25, 0.5, or 0.75 mg/mL of PZH was 56.6 ± 5.6%, 44.5 ± 5.3%, 45.1 ± 7.9%, or 26.6 ± 6.1%, respectively (Figure 5, *P* < 0.05).

### 3.5. PZH Suppresses MDR-Induced Activation of TGF-*β* Pathway in HCT-8/5-FU Cells

**Figure 6:**
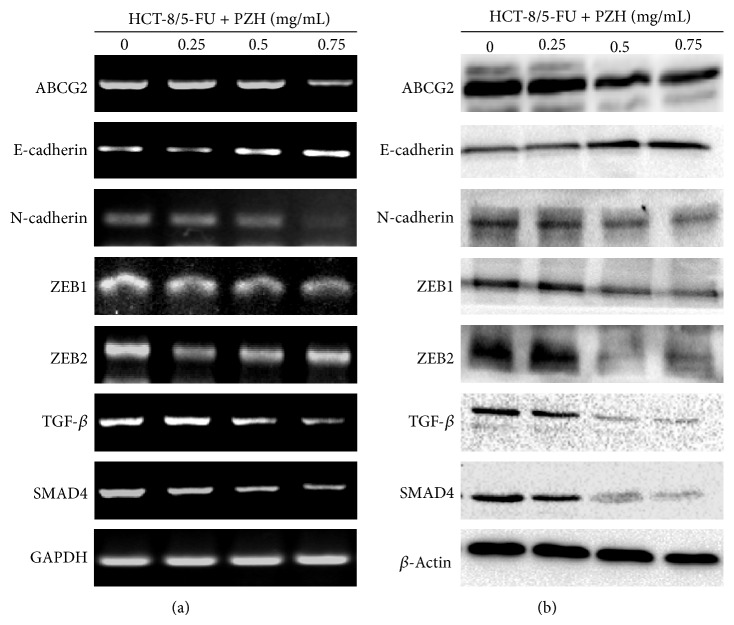
Effect of PZH on the expression of ABCG2, EMT-related factors, and activation of TGF-*β* pathway in HCT-8/5-FU cells. HCT-8/5-FU cells were treated with indicated concentrations of PZH for 24 h. The mRNA (a) and protein (b) expression levels of ABCG2, E-cadherin, N-cadherin, ZEB1, ZEB2, TGF-*β*, and SMAD4 in HCT-8/5-FU cells were determined by RT-PCR and Western Blot analyses. GAPDH or *β*-actin was used as the internal control for RT-PCR or Western Blot, respectively. Images are representatives of three independent experiments.

To explore the underlying mechanisms of inhibitory activities of PZH on cancer MDR and EMT, we determined its effect on the activation of the TGF-*β* pathway. As shown in Figure 3(b), acquisition of drug resistance significantly increased the mRNA expression of TGF-*β* and SMAD4 in HCT-8 cells, which however was suppressed by PZH treatment in a dose-dependent manner (Figure 6(a)). The protein expression patterns of TGF-*β* and SMAD4 in different cell groups were similar to the patterns observed for the respective mRNA (Figures 3(c) and 6(b)), suggesting that PZH inhibits colorectal cancer cell MDR/EMT probably through suppression of TGF-*β* signaling pathway.

## 4. Discussion

Multidrug resistance (MDR) and toxicity profoundly limit the effectiveness of currently used chemotherapeutic regimens for many human malignancies including colorectal cancer (CRC) [5–8]. Thus, the development of safer therapeutic agents with the ability to overcome the MDR could be a promising strategy to improve the effectiveness of anticancer therapies. Traditional Chinese medicines (TCM) have received recent interest since they have relatively few side-effects as compared to modern chemotherapeutics and have been used for thousands of years to clinically treat various diseases including cancer. Pien Tze Huang (PZH), a well-known TCM formula first prescribed in the Ming Dynasty, has long been used as a folk remedy for cancers in China and Southeast Asia. We recently demonstrated that PZH suppresses colorectal cancer growth* in vivo* and* in vitro* via modulation of multiple CRC-related signaling pathways, promotion of cancer cell apoptosis, and inhibition of cell proliferation and tumor angiogenesis [42–47]. These data suggest that PZH possesses a broad range of anticancer activities due to its ability to affect multiple intracellular targets. To further elucidate the antitumor mechanisms of PZH, in the present study we used a 5-FU resistant human colorectal carcinoma cell line (HCT-8/5-FU) to evaluate PZH's effects on drug resistance. We found that PZH significantly and dose-dependently inhibits the viability of 5-FU and ADM resistant HCT-8/5-FU cells. Moreover, PZH increased the accumulation of Rhodamine-123 and downregulated the drug resistance induced expression of ABCG2 in HCT-8/5-FU cells, thus demonstrating the inhibitory effect of PZH on chemoresistance of colorectal cancer cells.

Since the process of epithelial-mesenchymal transition (EMT) plays important roles in drug resistance [25–33], we examined the effect of PZH on EMT in HCT-8/5-FU cells. Consistent with previous studies that MDR induces EMT-related properties in cancer cells [48], we observed that acquisition of drug resistance in HCT-8 cells resulted in EMT-related morphological changes (e.g., loss of cell polarity, spindle-shaped fibroblastoid-like morphology, and formation of pseudopodia) and alteration in the expression of EMT-regulatory factors, including the decrease of epithelial marker E-cadherin expression and the upregulation of expression of mesenchymal markers such as N-cadherin, ZEB1, and ZEB2. However, these EMT-related changes in HCT-8/5-FU cells were significantly neutralized by PZH treatment. Moreover, we found that drug resistance significantly enhanced the capacities of migration and invasion in colorectal cancer cells. PZH treatment significantly inhibited the migration and invasion of HCT-8/5-FU cells in a dose-dependent manner. Cancer EMT is mediated by multiple intracellular pathways including TGF-*β* signaling. To further investigate the mechanisms of inhibitory activities of PZH on cancer MDR and EMT, we determined its effect on the activation of TGF-*β* signaling pathway. As expected, acquisition of drug resistance significantly increased mRNA and protein expression levels of TGF-*β* and SMAD4. However, MDR-induced upregulation of TGF-*β* and SMAD4 expression was significantly suppressed by PZH treatment, suggesting that PZH suppresses TGF-*β* pathway in drug-resistant colorectal cancer cells.

In conclusion, we demonstrate for the first time that PZH can effectively overcome MDR and inhibit EMT in CRC cells via suppression of the TGF-*β* pathway. Together with our previous studies that PZH suppresses the activation of several CRC-related signaling pathways including STAT3, Akt, and MAPKs, we speculate that PZH may exert its inhibitory activities on MDR and EMT in a holistic way by modulating multiple cellular signal transduction pathways. This intriguing observation should be addressed in future studies to fully elucidate the molecular mechanism of the tumoricidal activity of PZH, which may help to develop better multitarget drugs for cancer therapy.

## Figures and Tables

**Table 1:** Sequence of primers (5′ to 3′).

ABCG2	Sense: GCCGTGGAACTCTTTGTGGTAG
	Antisense: ACAGCAAGATGCAATGGTTGT

E-cadherin	Sense: CTACAATGCCGCATCGCTT
	Antisense: GTATACGTAGGGAAACTCTCTCGGTC

N-cadherin	Sense: AAGAACGCCAGGCCAAACAAC
	Antisense: CTGGCTCAAGTCATAGTCCTGGTCT

ZEB1	Sense: AAGAATTCACAGTGGAGAGAAGCCA
	Antisense: CGTTTCTTGCAGTTTGGGCATT

ZEB2	Sense: TGTCATTAGAAGAGGCGTAA
	Antisense: GCAGAGCAGGTTAGAACT

TGF-*β*	Sense: ACCCACAACGAAATCTATGACA
	Antisense: CTAAGGCGAAAGCCCTCAAT

Smad4	Sense: GATTTGCGTCAGTGTCATCG
	Antisense: AGTCTAAAGGTTGTGGGTCTG

GAPDH	Sense: GTCATCCATGACAACTTTGG
	Antisense: GAGCTTGACAAAGTGGTCGT

## Abbreviations

PZH: Pien Tze Huang
TCM: Traditional Chinese medicine
CRC: Colorectal cancer
MDR: Multidrug resistance
EMT: Epithelial-to-mesenchymal transition
TGF-*β*: Transforming growth factor *β*.

## References

[1] Jemal A., Bray F., Center M. M., Ferlay J., Ward E., Forman D. (2011). Global cancer statistics. *CA: A Cancer Journal for Clinicians*.

[2] Markowitz S. D., Bertagnolli M. M. (2009). Molecular basis of colorectal cancer. *The New England Journal of Medicine*.

[3] Cunningham D., Atkin W., Lenz H.-J., Lynch H. T., Minsky B., Nordlinger B., Starling N. (2010). Colorectal cancer. *The Lancet*.

[4] Jiang W. Q., Fu F. F., Li Y. X., Wang W. B., Wang H. H., Jiang H. P., Teng L. S. (2012). Molecular biomarkers of colorectal cancer: prognostic and predictive tools for clinical practice. *Journal of Zhejiang University: Science B*.

[5] Van Cutsem E., Costa F. (2005). Progress in the adjuvant treatment of colon cancer: has it influenced clinical practice?. *Journal of the American Medical Association*.

[6] Longley D. B., Allen W. L., Johnston P. G. (2006). Drug resistance, predictive markers and pharmacogenomics in colorectal cancer. *Biochimica et Biophysica Acta*.

[7] Lippman S. M. (2006). The dilemma and promise of cancer chemoprevention. *Nature Clinical Practice Oncology*.

[8] Lin L., Liu Y., Li H., Li P.-K., Fuchs J., Shibata H., Iwabuchi Y., Lin J. (2011). Targeting colon cancer stem cells using a new curcumin analogue, GO-Y030. *British Journal of Cancer*.

[9] Gottesman M. M., Fojo T., Bates S. E. (2002). Multidrug resistance in cancer: role of ATP-dependent transporters. *Nature Reviews Cancer*.

[10] Saha S., Adhikary A., Bhattacharyya P., Das T., Sa G. (2012). Death by design: where curcumin sensitizes drug-resistant tumours. *Anticancer Research*.

[11] Bhangu A., Wood G., Mirnezami A., Darzi A., Tekkis P., Goldin R. (2012). Epithelial mesenchymal transition in colorectal cancer: seminal role in promoting disease progression and resistance to neoadjuvant therapy. *Surgical Oncology*.

[12] Zavadil J., Cermak L., Soto-Nieves N., Böttinger E. P. (2004). Integration of TGF-*β*/Smad and Jagged1/Notch signalling in epithelial-to-mesenchymal transition. *The EMBO Journal*.

[13] Pérez-Tomás R. (2006). Multidrug resistance: retrospect and prospects in anti-cancer drug treatment. *Current Medicinal Chemistry*.

[14] Glavinas H., Krajcsi P., Cserepes J., Sarkadi B. (2004). The role of ABC transporters in drug resistance, metabolism and toxicity. *Current Drug Delivery*.

[15] Zhu M. M., Tong J. L., Xu Q., Nie F., Xu X. T., Xiao S. D., Ran Z. H. (2012). Increased JNK1 signaling pathway is responsible for ABCG2-mediated multidrug resistance in human colon cancer. *PLoS ONE*.

[16] Huang W.-C., Hsieh Y.-L., Hung C.-M., Chien P.-H., Chien Y.-F., Chen L.-C., Tu C.-Y., Chen C.-H., Hsu S.-C., Lin Y.-M., Chen Y.-J. (2013). BCRP/ABCG2 inhibition sensitizes hepatocellular carcinoma cells to sorafenib. *PLoS ONE*.

[17] Thiery J. P., Sleeman J. P. (2006). Complex networks orchestrate epithelial-mesenchymal transitions. *Nature Reviews Molecular Cell Biology*.

[18] Nieto M. A. (2002). The snail superfamily of zinc-finger transcription factors. *Nature Reviews Molecular Cell Biology*.

[19] Peinado H., Olmeda D., Cano A. (2007). Snail, ZEB and bHLH factors in tumour progression: an alliance against the epithelial phenotype?. *Nature Reviews Cancer*.

[20] Thiery J. P., Acloque H., Huang R. Y. J., Nieto M. A. (2009). Epithelial-mesenchymal transitions in development and disease. *Cell*.

[21] Kalluri R., Weinberg R. A. (2009). The basics of epithelial-mesenchymal transition. *Journal of Clinical Investigation*.

[22] Turley E. A., Veiseh M., Radisky D. C., Bissell M. J. (2008). Mechanisms of disease: epithelial-mesenchymal transition—does cellular plasticity fuel neoplastic progression?. *Nature Clinical Practice Oncology*.

[23] Huber M. A., Kraut N., Beug H. (2005). Molecular requirements for epithelial-mesenchymal transition during tumor progression. *Current Opinion in Cell Biology*.

[24] Bates R. C., Mercurio A. M. (2005). The epithelial-mesenchymal transition (EMT) and colorectal cancer progression. *Cancer Biology & Therapy*.

[25] Polyak K., Weinberg R. A. (2009). Transitions between epithelial and mesenchymal states: acquisition of malignant and stem cell traits. *Nature Reviews Cancer*.

[26] Rosanò L., Cianfrocca R., Spinella F., di Castro V., Nicotra M. R., Lucidi A., Ferrandina G., Natali P. G., Bagnato A. (2011). Acquisition of chemoresistance and EMT phenotype is linked with activation of the endothelin A receptor pathway in ovarian carcinoma cells. *Clinical Cancer Research*.

[27] Arumugam T., Ramachandran V., Fournier K. F., Wang H., Marquis L., Abbruzzese J. L., Gallick G. E., Logsdon C. D., McConkey D. J., Choi W. (2009). Epithelial to mesenchymal transition contributes to drug resistance in pancreatic cancer. *Cancer Research*.

[28] Singh A., Settleman J. (2010). EMT, cancer stem cells and drug resistance: an emerging axis of evil in the war on cancer. *Oncogene*.

[29] Kajiyama H., Shibata K., Terauchi M., Yamashita M., Ino K., Nawa A., Kikkawa F. (2007). Chemoresistance to paclitaxel induces epithelial-mesenchymal transition and enhances metastatic potential for epithelial ovarian carcinoma cells. *International Journal of Oncology*.

[30] Shah A. N., Summy J. M., Zhang J., Park S. I., Parikh N. U., Gallick G. E. (2007). Development and characterization of gemcitabine-resistant pancreatic tumor cells. *Annals of Surgical Oncology*.

[31] Işeri Ö. D., Kars M. D., Arpaci F., Atalay C., Pak I., Gündüz U. (2011). Drug resistant MCF-7 cells exhibit epithelial-mesenchymal transition gene expression pattern. *Biomedicine and Pharmacotherapy*.

[32] McConkey D. J., Choi W., Marquis L. (2009). Role of epithelial-to-mesenchymal transition (EMT) in drug sensitivity and metastasis in bladder cancer. *Cancer and Metastasis Reviews*.

[33] Yang A. D., Fan F., Camp E. R. (2006). Chronic oxaliplatin resistance induces epithelial-to-mesenchymal transition in colorectal cancer cell lines. *Clinical Cancer Research*.

[34] Willis B. C., Borok Z. (2007). TGF-*β*-induced EMT: mechanisms and implications for fibrotic lung disease. *American Journal of Physiology—Lung Cellular and Molecular Physiology*.

[35] Xu J., Lamouille S., Derynck R. (2009). TGF-*β*-induced epithelial to mesenchymal transition. *Cell Research*.

[36] Allendorph G. P., Read J. D., Kawakami Y., Kelber J. A., Isaacs M. J., Choe S. (2011). Designer TGF*β* superfamily ligands with diversified functionality. *PLoS ONE*.

[37] Moustakas A., Heldin C.-H. (2007). Signaling networks guiding epithelial-mesenchymal transitions during embryogenesis and cancer progression. *Cancer Science*.

[38] Wu Y., Sato F., Yamada T., Bhawal U. K., Kawamoto T., Fujimoto K., Noshiro M., Seino H., Morohashi S., Hakamada K., Abiko Y., Kato Y., Kijima H. (2012). The BHLH transcription factor DEC1 plays an important role in the epithelial-mesenchymal transition of pancreatic cancer. *International Journal of Oncology*.

[39] Gordaliza M. (2007). Natural products as leads to anticancer drugs. *Clinical and Translational Oncology*.

[40] Ji H.-F., Li X.-J., Zhang H.-Y. (2009). Natural products and drug discovery. *EMBO Reports*.

[41] Chinese Pharmacopoeia Commission (2010). *Pharmacopoeia of the Peoples Republic of China*.

[42] Lin J.-M., Wei L.-H., Chen Y.-Q., Liu X.-X., Hong Z.-F., Sferra T. J., Peng J. (2011). Pien Tze Huang-induced apoptosis in human colon cancer HT-29 cells is associated with regulation of the Bcl-2 family and activation of caspase 3. *Chinese Journal of Integrative Medicine*.

[43] Zhuang Q., Hong F., Shen A., Zheng L., Zeng J., Lin W., Chen Y., Sferra T. J., Hong Z., Peng J. U. N. (2012). Pien Tze Huang inhibits tumor cell proliferation and promotes apoptosis via suppressing the STAT3 pathway in a colorectal cancer mouse model. *International Journal of Oncology*.

[44] Shen A.-L., Hong F., Liu L.-Y., Lin J.-M., Zhuang Q.-C., Hong Z.-F., Peng J. (2012). Effects of Pien Tze Huang on angiogenesis in vivo and in vitro. *Chinese Journal of Integrative Medicine*.

[45] Shen A., Hong F., Liu L., Lin J., Wei L., Cai Q., Hong Z., Peng J. (2012). Pien Tze huang inhibits the proliferation of human colon carcinoma cells by arresting G1/S cell cycle progression. *Oncology Letters*.

[46] Shen A., Chen Y., Hong F., Lin J., Wei L., Hong Z., Sferra T. J., Peng J. (2012). Pien Tze Huang suppresses IL-6-inducible STAT3 activation in human colon carcinoma cells through induction of SOCS3. *Oncology Reports*.

[47] Shen A., Lin J., Chen Y. (2013). Pien Tze Huang inhibits tumor angiogenesis in a mouse model of colorectal cancer via suppression of multiple cellular pathways. *Oncology Reports*.

[48] Pasqualato A., Palombo A., Cucina A., Mariggiò M. A., Galli L., Passaro D., Dinicola S., Proietti S., D'Anselmi F., Coluccia P., Bizzarri M. (2012). Quantitative shape analysis of chemoresistant colon cancer cells: correlation between morphotype and phenotype. *Experimental Cell Research*.

